# Prescription Department

**Published:** 1894-06

**Authors:** 


INFANT LAXATIVE MIXTURE.

℞ Nitrat. potass., gr. j.

Sulph. magnes., gr. v.

Syr. lemon, ʒ ss.

Water, q. s. ʒ j.

Mix. Dose, ʒ j.—*Roosevelt Hospital Formulary; The Prescription.*

CHILDREN'S TONIC.

℞ Ammon. cit. iron, gr. v.

Sol. arsenit. potass., *m* ij.

Syr. orange, ʒ ss.

Water, q. s. ʒ j.

Mix. Dose, ʒ j.—*Ibid.*

GONORRHEA.

As an injection Waltier prefers:

℞ Hydrarg. chlorid. corros., gr. ⅛.

Antipyrin., ʒ ss.

Aq. destil., ℥ vj.

M. Four injections daily.

Internally, in order to prevent painful erections, he gives every night 15 grains of antipyrin and 45 grains of bromide of potassium.—*La Reforma Medica.*

ANOREXIA.

For loss of appetite with inanition, in children, the *Hot Springs Medical Journal* recommends:
℞ Acid. phosphoric. dil., *m* xxxij.
Tinct. nucis vomicæ, *m* viij.
Vin. xerici,
Syr. lactopeptin. vel. simplicis,āā ʒ iv.
Aquæ, q. s. ad. ℥ ij.

M. Sig. Take 1 teaspoonful three times a day, before meals. For a child two years old, give one teaspoonful of this mixture ten minutes before meals.

NASO-PHARYNGEAL CATARRH OF INFANTS.

M. H. Neumann recommends:
℞ Zinc sulphate, gr. iss.
Water, f ℥ ss.

Instill into each nostril a few drops of this solution several times daily.—*American Medico-Chirurgical Bulletin.*

ASTHMA.

℞ Etheris, ℥ j.

Ol. terebinth., ʒ iij.

Acid. benzoic., ʒ iij.

Balsam. tolutan., ʒ ij.—M.

For inhalation during paroxysm:

℞ Sulphate of strychnine, gr. 1/8–1/4.
Powdered ipecacuanha, gr. ivss.
Powdered black pepper, gr. ivss.
Extract of gentian, gr. xx.
Essence of periwinkle, gtt. j.

Mix and make 20 pills.

Sig. A pill after each meal when asthma depends upon digestive trouble.

℞ Arsenious acid, gr. j.
Hydrochlorate of quinine, gr. xv.
Sulphate of atropine, gr. ss.
Extract of gentian, q. s.

Mix and make 60 pills.

Sig. Take four pills daily.

*Lebert in Rev. Intern. de Rhinologie, etc.*

SALINE LAXATIVE.

Constantin Paul recommends phosphate of sodium as a substitute for sulphate of sodium. He gives it in the form of lemonade. It does not gripe. The following formulæ are used:

℞ Distilled water, ℥ vj.

Phosphate of sodium, ʒ vi½.

Essence of citron, gtt. xx.

Syrup, ℥ ij.—M.

℞ Distilled water, ℥ viij.

Phosphate of sodium, ʒ vi½.

Essence of citron, gtt. xxv.

Syrup, ℥ ij.

Citric acid,

Bicarbonate of sodium, āā ʒ iss.—M.

An effervescing mixture.—*Le Prog. Méd.*

FOR SORE THROAT.

℞ Potass chlorates,

Aluminis, āā ʒ ss.

Aq. puræ, ℥ iv.

Millis, ℥ iss.

Tinct. myrrh, ℥ ss.

Listerine, ℥ ss.

M. Sig. Use as a gargle every two or three hours.—*Ex.*

